# Lifestyle, Exercise and Activity Package for People living with Progressive Multiple Sclerosis (LEAP-MS): adaptions during the COVID-19 pandemic and remote delivery for improved efficiency

**DOI:** 10.1186/s13063-021-05245-1

**Published:** 2021-04-16

**Authors:** Rachel Lowe, Christy Barlow, Barry Lloyd, Julie Latchem-Hastings, Vincent Poile, Charlotte Scoble, Andrew Dean-Young, Kate Button, Rebecca Playle, Monica Busse

**Affiliations:** 1 Centre for Trials Research, Cardiff University, 4th floor Neuadd Meirionnydd, Heath Park, Cardiff, UK; 2 School of Healthcare Sciences, Cardiff University, Ty Dewi Sant, Heath Park, Cardiff, UK

**Keywords:** COVID-19 adaptions, Trial management, Recruitment, Electronic consent, Feasibility study, Remote intervention delivery, Multiple sclerosis, Physiotherapy, Physical activity, Self-management

## Abstract

**Abstract:**

The LEAP-MS (Lifestyle, Exercise and Activity Package for People living with Progressive Multiple Sclerosis) study has developed an individualised supported self-management approach for physical activity for people with progressive multiple sclerosis (MS) and severe disability. The intervention has been evaluated in a single-arm feasibility study with embedded process evaluation. The feasibility study was due to open to recruitment during the COVID-19 2020–2021 pandemic, 1 month into the first UK-wide lockdown. We worked rapidly to implement adaptions to the trial procedures and intervention delivery that we believe are applicable to randomised controlled trials.

Recruitment became predominantly via self-referral. Electronic consent was employed, with consent discussions occurring over the telephone. Registration, consent, eligibility assessment and data collection as well as the intervention (online physical activity tool) were via a secure, encrypted multi-user web-based platform for participants, physiotherapists and researchers accessible via various hardware. Physiotherapy consultations, as well as the process evaluation, were conducted remotely using video conferencing software or the telephone. A remote training package for physiotherapists and site initiations was also developed and electronic site files employed.

Our adaptions are extremely topical given the COVID-19 situation, and whilst not what we had originally planned, have enabled successful delivery of the feasibility study and are relevant to conducting randomised controlled trials and meeting the needs of people with MS who are far more isolated than ever before.

**Trial registration:**

ClinicalTrials.govNCT03951181. Registered on 15 May 2019.

## Background to LEAP-MS

LEAP-MS (Lifestyle, Exercise and Activity Package for People living with Progressive Multiple Sclerosis) aims to develop and evaluate an individualised supported self-management approach for physical activity with a specific focus on people with progressive multiple sclerosis (PwPMS) and severe disability.

Multiple sclerosis (MS) is a long-term deteriorating condition which causes a range of symptoms like blurred vision and problems with how people move, think and feel affecting an estimated 107,000 people in the UK [1]. Of these it is estimated that 10–15,000 have primary progressive MS [2] and 38,000 secondary progressive MS [3], which are characterised by worsening of symptoms either independent of relapses/remissions or with relapses. PwPMS often have higher levels of disability than those with relapsing-remitting MS, often have high health and social care needs, and self-report low health-related quality of life [4, 5].

Regular physical activity is generally regarded to be an important component of long-term management of MS [6, 7]. Physical activity may have a positive impact on MS symptoms, including mobility, cognition and fatigue, and have important physical, psychological and social benefits [8, 9]. Various interventions are reported in the literature, ranging from group interventions to digital versatile disk (DVD) and web-based interventions [10–14] but most research to date has focussed on patients who are walking and evidence in progressive MS is inconclusive [8, 15, 16].

Despite the possible benefits, we know that people with MS want to be physically active [6, 17]. In particular, those who are more disabled find it hard to start and maintain activity, and often do not receive any or enough support [18]. Pro-active approaches to supporting physical activity (often called self-management interventions), that empower individuals to continue to be physically active independently following health professional input are therefore urgently needed for this group of people with MS [19].

In MS there is an additional need for health professionals to really understand the condition and have the knowledge and skills in relation to self-management of physical activity [19]. As supporting people to self-manage their physical activity might require physiotherapists to work differently, we need to know more about the specific training they need to provide the right sort of support. We also need to know more about how physical activity interventions can be successfully personalised to meet the needs of individuals with PwPMS, and what the associated costs/challenges are to the use of this approach in practice.

### Phase 1: qualitative interview study

In phase 1 of the LEAP-MS study, we collected information about the barriers to and facilitators of physical activity that PwPMS experience, their current levels and type of physical activity and their perceptions of the role physical activity plays in managing MS symptoms from both them and their families—or people that support them. This provided us with important information about why physical activity might be important for PwPMS, the challenges they face in doing physical activity or accessing it, and ways which they have found to overcome any barriers. We also collected information from physiotherapists about their understanding of self-management and their needs for training about using self-management approaches with PwPMS.

### Phase 2: intervention development and feasibility testing

We used this information gathered in Phase 1 to work with PwPMS and physiotherapists to co-design the LEAP-MS personalised intervention to facilitate on-going physical activity for people with PwPMS. The outcomes achieved through the collaboration have exceeded expectations through the co-production of an online education and activity platform, a patient-led approach to consultations, an online hub providing resources for clinicians delivering research protocols and a novel online-only evaluation method (paperless) for clinical evaluation of the co-produced interventions.

The intervention is made up of:

- A multi-user web-based online physical activity tool
- Up to six physiotherapy consultations (coaching sessions)
- A training package for physiotherapists about self-management with PwPMS

Further details of the intervention development are published separately [20].

We sought to evaluate the feasibility and acceptability of the intervention and trial procedures in a single-arm feasibility study with embedded process evaluation [21]. The study was due to open to recruitment in April 2020, 1 month into the first UK wide lockdown during the COVID-19 2020–2021 pandemic [22].

### Original feasibility study protocol (pre-COVID-19 pandemic)

The study had ethical approval (Wales REC 6, reference 19/WA/0195) and sought R&D approval from three NHS Health Boards in Wales to recruit 21 participants via three recruitment routes, namely (1) a MS research database hosted in the NHS; (2) referral from NHS outpatient physiotherapy services; (3) self-referral from within Health Boards. Recruitment routes 1 and 3 required the potential participant to register their interest in the study via the LEAP-MS website, self-complete an online consent form, initial eligibility screen and baseline self-completion measures. For participants recruited via route 2 in physiotherapy outpatient clinics, written informed consent, eligibility assessment and baseline measures would be undertaken at the clinic in person. In all cases, consent and eligibility would be reconfirmed at the initial home-based (face-face) coaching session by a physiotherapist, who had received tailored intervention and study procedure training. Eligible patients had either primary or secondary progressive multiple sclerosis (as defined by the Lublin classification) [23], were aged 18 or over and had an Expanded Disability Status Scale (EDSS) score [24] between six (able to walk 100 m with an aid) and eight (restricted to bed, chair or wheelchair but may be out of bed much of the day). Participants had capacity to consent to study participation on their own behalf and had access to mobile, wireless or wired internet connection at home. Any individuals with relapsing-remitting or non-progressive MS, were unable to understand written and spoken English, or were pregnant or planning a pregnancy were excluded.

Following the initial coaching session, the participants would have been given access to the online intervention for an initial 3-month period. During this period participants would be able to request up to five further home-based physiotherapy coaching sessions, after which the LEAP-MS platform without physiotherapy support would be available to participants for a further 3 months. Follow-up was scheduled for 3-months and 9-months post-baseline. The following outcome measures were to be self-completed by the participants and collected using the online study platform at baseline and both follow-up timepoints:

- Modified form of the Fatigue Impact Scale (MFIS) [25] to assess fatigue in terms of physical, cognitive, and psychosocial functioning;
- Multiple Sclerosis Impact Scale (MSIS-29) to measure the physical and psychological impact of MS from the patient’s perspective [26];
- EQ-5D-5 L to provide an indication of health-related quality of life [27];
- Oxford Participation and Activities Questionnaire (OxPAQ) to assess the impact of ill-health on participation, activities and autonomy [28];
- University of Washington 6-item short form self-efficacy scale (UW-SES-SF) (MS specific) to indicate self-efficacy [29];
- Modified Patients’ Global Impression of Change (PGIC) [30] at 3 months and 9 months follow-up only.

Semi-structured interviews were going to be conducted in person with participants 3 months after baseline. Consenting intervention physiotherapists were also going to be interviewed in person once all their participants had received the intervention for the initial 3-month period. Usage of the LEAP-MS platform was going to be tracked during the 6-month intervention period. Intervention physiotherapy notes were intended to be completed on paper, as was the eligibility assessment undertaken by the physiotherapist and any safety reporting case report forms. The intervention coaching sessions were going to be observed in person by the study’s qualitative researcher. Costs associated with intervention delivery (therapist travel and contact time) were also going to be recorded by the physiotherapists.

### COVID-19 pandemic restrictions and implications

The COVID-19 2020/2021 pandemic [22] and the resulting imposed restrictions [31, 32] meant that many of the intended study procedures described above were not feasible. Recruitment into clinical trials and studies that were not related to COVID-19 ceased within the NHS [33]. NHS staff who had been trained to deliver the intervention had to be redeployed to hospital wards or in community positions and were unable to deliver the intervention as planned [34, 35]. Exercise and social groups were disbanded, and gyms and leisure centres closed [31, 32]. Social distancing rules prevented face-face visits being conducted. Obtaining paper-based written informed consent and paper-based data collection also became problematic.

## Revised feasibility study protocol

Given the impending start of recruitment we worked rapidly to implement amendments and succeeded in securing a 6-month no-cost extension from the funder (MS Society). The following amendments to the feasibility study protocol were classed as non-substantial amendments under the guidance issued by the Health Research Authority [36] and the revised protocol was submitted for publication (currently under review) [21].

### Research sites

As the intended research sites were unable to open to recruitment due to staff redeployment and revised NHS priorities, non-NHS research sites were set up. Physiotherapists were utilised from within the project team to deliver the intervention, so not to draw resources from the NHS. NHS research sites were able to open to recruitment once recruitment to non-COVID-19 clinical trials and studies resumed.

### Training

Time was invested in developing a bespoke LEAP-MS physiotherapy training package that focused on the provision of self-management support to participants, the use of technology in consultations, updates on physical activity and exercise guidelines for long-term neurological conditions, what to expect when using the LEAP platform along with potential challenges and solutions. The initial training package was developed through a two-day face-face interactive workshop underpinned by Bridges Self-Management principles and delivered by Bridges Social Enterprise (http://www.bridgesselfmanagement.org.uk/), in which ten physiotherapists participated in prior to the COVID-19 pandemic. The workshop was video recorded and utilised as part of the final online training package. Further resources to help structure remote interactions (https://www.bridgesselfmanagement.org.uk/covid-19-resources/) were made available to standardise coaching interactions regardless of mode of delivery. Site initiation training in study procedures was delivered remotely via Zoom [37], recorded and made available as part of the online training package. User guides on study processes were also developed. Physiotherapists were given access to conversation-based scripts to guide coaching conversations and had the opportunity to practice coaching conversations and receive peer review. All resources were accessed via the LEAP-MS multimedia online learning resource.

### Recruitment, e-consent and eligibility assessment

Physiotherapists who attended the intervention training and site initiation were emailed the physiotherapist information sheet and a link to the online consent form. They were asked to complete an informed consent form using Cardiff online survey software (https://cardiff.onlinesurveys.ac.uk/), save a local copy of the consent form and email it to the LEAP-MS email address. A copy was also saved in the electronic investigator site file.

MS Society endorsed communication channels were utilised as an additional recruitment pathway. Potential participants within the South Wales region were emailed by the MS register. The MS research database was also used as initially planned, from which potential participants were sent a letter. Both the email and letter were accompanied by the patient information sheet. Potential participants then registered their interest using the online study platform generating a unique login, providing their contact details and self-completing a brief online eligibility screen. An automated email was sent to LEAP-MS email account to notify the study team of the registration. After which, the participant was contacted by telephone by the central study team, given the opportunity to ask questions and have their responses to the initial eligibility screen reviewed. If deemed to still be eligible, the electronic consent form was released to the potential participant’s LEAP-MS account through the online platform and they were also emailed to request they login using their registered login and complete the online consent form and the baseline measures. The e-consent form consisted of declarations with yes/no tick boxes, typed name, typed date and date of birth, and an automatic date/time stamp generated as part of the audit trail upon saving the form. Eligibility was reconfirmed by the intervention physiotherapist during the initial online (Zoom) coaching session and recorded by the physiotherapist on an electronic case report form accessed from the online platform.

### Intervention delivery

Virtual physiotherapy consultations using web conferencing software (Zoom [37]) or telephone were allowed in addition to, or in replace of, face-face consultations. Participants were offered a practice Zoom session with the central study team prior to the first physiotherapist consultation (coaching session) to facilitate familiarisation with the technology. We also tried to balance the assignment of physiotherapists to cover different environments (NHS based and University based, different levels of training and different referral routes). All risk assessment documentation was revised to address remote intervention delivery.

### Data collection and management

The follow-up period was reduced from 9-months to 6-months and the economic evaluation was removed to streamline data collection.

We developed a secure, encrypted multi-user web-based platform for participants, physiotherapists and researchers accessible via desktop computers, laptops, tablets or smart phones. Participants were able to use the platform to register, complete eligibility forms, consent, baseline and follow-up measures as well as to access the intervention. The physiotherapist-completed case report forms were incorporated into the online platform, with the added functionality of being able to download the therapy notes to facilitate incorporation into the patients’ NHS medical records. Participants, physiotherapists and administrators all had different access and editing level permissions. As participants entered their own data most of the data could not be queried; to limit errors and ensure quality data inbuilt database validations were used including using input masks, restricted data formats and mandatory data fields. Effort was made to make the platform visually appealing, easy to navigate and allowed one measure/case report form to be completed and saved at a time. The platform was also used by the study team to evaluate participant engagement with the intervention and to manage study data.

The process evaluation also had to be undertaken remotely; recorded Zoom coaching sessions were used for the observations and the qualitative interviews were conducted over Zoom or telephone.

The use of an electronic study master file and electronic investigator site files were also employed. Microsoft One Drive and password protected files on secure shared drives on University servers were used.

## Discussion: efficiencies and challenges

Efficiencies have been achieved in multiple areas relevant to the delivery of the LEAP-MS study. Most notably flexibility in intervention delivery reduced burden on the NHS and ensured that we were able to successfully recruit, consent, deliver assessments and evaluate outcomes in a group of PwPMS who were increasingly isolated given the COVID-19 lockdown restrictions. In our view, the availability of an online training package and the conduct of remote site initiation was key to the success we observed in rapidly opening sites. Some of the physiotherapists who were relatively new to research required additional assistance and support when undertaking the study processes. This may not have been the case had training been delivered face-face. Whilst remote training may reduce site buy-in and rapport due to the limited personal contact, we would recommend that the mode of delivering the training and site initiations should be considered on a study-by-study basis.

Advertising the study to potential participants via MS Society communication channels proved highly efficient providing greater reach as expressions of interest became independent of Health Board and gave scope for increased generalisability. It was however limited in that the study team were not able to ensure clinical confirmation of the EDSS score. This was dealt with through an additional eligibility screen conducted by the central study team followed by a further physiotherapist eligibility assessment at the first physiotherapy consultation.

We used an electronic trial master file and site files and developed a system to enable the electronic registration and consent process. A separate study visit was thus not required to obtain informed consent thereby reducing both staff and participant burden. We found that approximately a third of participants required explicit instructions and a link to the website/consent form area, before they were successfully able to access the website and complete the online consent form. This may have been compounded by the patient population as impaired cognitive and memory function are common symptoms of MS; however, we would recommend building an automated email function into the online platform to notify participants when the consent form is released to them for completion.

The LEAP-MS database acts as the source consent documentation and will be retained for the required archiving period in line the requirements for archiving the electronic data set, and thereby also being accessible in case of a statutory inspection(s). The LEAP-MS patient information sheet explained that University staff would have access to their data and the completed consent form, and explicit consent was sought for this as one of the statements on the consent form. A copy of the completed consent form was downloaded and saved in the electronic investigator site file for retention by the site. The participant had access to their completed consent form through the online platform. We are considering how to enable the participant to have long-term access to the completed consent form. The intention for future iterations of the platform is to enable participants to download, save and/or print their completed consent form. For this study, we intend to download and post the completed consent form to the participant’s home so they have a copy once access to the platform is removed.

The e-consent process and system used in LEAP-MS exceeds the ethical and legal requirements for e-consent recommended for ‘other types of research’ as described in the joint statement on seeking consent by electronic methods [38]; it also fulfils the e-consent requirements for type A Clinical Trials of Investigational Medicinal Products (CTIMPs) [39]. For higher risk trials, the verification process could be strengthened by checking identification (e.g. passport or driving licence) as part of the video conference call. We believe there is potential to utilise this approach to collect baseline data from the participant prior to a study visit where consent can be confirmed in person prior to the administration of any Investigation Medicinal Product or higher-risk trial intervention.

Due to the COVID-19 pandemic there has been an expedited move towards telemedicine and remotely delivered rehabilitation [40, 41]. This trend looks set to continue and we are likely to see many changes in the delivery of rehabilitation moving forward [42, 43]. Most trials that are delivered remotely still include face-face components [44, 45]. The LEAP-MS intervention was originally designed to have face-face coaching sessions alongside a web-based platform. The existing platform facilitated modifications to remote delivery only. Crucial to moving to remote delivery were meeting the additional training needs of both the physiotherapists and the participants, as well as considering how to conduct the fidelity evaluation. Physiotherapists and participants required some level of digital literacy to take part in LEAP-MS. Physiotherapists required practice sessions using both the online platform and the video conferencing software, particularly when using them simultaneously, and for some participants, LEAP-MS was their first experience of using online conferencing and an online resource of any type. We would recommend time for this is built into the study timelines.

Technical issues were experienced in a number of consultations relating to internet connectivity, sound and video quality as well as user capability and confidence; two participants ended up having their first consultation over the telephone, one of these participants also had their second consultation over the phone. Attend Anywhere [46] was initially considered for the video conferencing software as it was rising in popularity within the NHS. Microsoft Teams [47] was also considered, however, we decided to use Zoom due to its ease of use (participant interface), recording ability (required for the process evaluation), and that the sponsor had a Zoom business account (additional confidence in data security). All the NHS intervention physiotherapists were given a University email account to enable them to use Zoom for their LEAP-MS consultations. Consideration of which video conferencing software to use in any future trial(s) will be required to maximise the benefit and minimise inconvenience.

Participants experienced a shorter waiting time (mean of 3 weeks) for their initial physiotherapy appointment compared to typical home visit waiting times of up to 8 weeks [48]. This was in part due to the study using both NHS and non-NHS physiotherapists but also because the physiotherapist’s time was maximised as travel to the participant’s home was not required and initial research activities such as expressions of interest, discussing and obtaining informed consent, and the initial eligibility screen had already been conducted prior to the first physiotherapy consultation.

The online intervention platform has been future-proofed in that recommendations for activities not allowed due to COVID-19 pandemic social restrictions have been blocked but were included in the build so that access can be easily allowed in future. LEAP-MS has also featured in the NIHR remote delivery guidance as an example of remote delivery of complex interventions [49].

Although this study is a single-arm feasibility study, the database has been developed so that it can be efficiently repurposed for a main randomised controlled trial. The participant self-report outcome measures used in LEAP-MS were intended to be collected remotely prior to the COVID-19 pandemic using the online platform. The database had in-built validations to ensure high-quality data and efficient data management. Limited data querying and data cleaning could be conducted as participants’ self-completed many of the measures. Data management tasks focused primarily on prompting participants to complete the forms. Initial prompts were conducted by email 2-weeks and 1-week before follow-ups were due, however, the number of completed forms improved when the email prompts were accompanied by telephone calls. This may in part be due to the patient population but could also be due to external factors effecting participants’ mood, such as the follow-ups coinciding with increased COVID-19 related social restrictions and the local and national lockdowns of autumn and winter 2020 [31, 32].

Some technical issues were experienced with the database, mainly that the data collection forms were locked part way through two participants completing their baseline measures, and three participants were able to complete their 6-month follow-up prior to their intended follow-up window. These errors were rectified as soon as the central team became aware. Although standard database testing was employed, the scheduling of access to forms was not initially tested, and this experience highlights the necessity for thorough testing prior to database release, especially when participants are entering their data themselves.

The physiotherapist-completed case report forms were incorporated into the online platform due to the COVID-19 implications. A benefit of this, in addition to reduced data entry and reduced printing and stationary costs, was the added functionality of being able to download the therapy notes. Although this function was not used during this study, it will facilitate incorporation into the patients NHS medical records in the future.

## Conclusion

In LEAP-MS, we have developed an entirely novel approach to remote participant enrolment, consent and assessment as well as a more comprehensive, interactive and adaptable intervention than was originally planned, which can be delivered remotely. Both of which are very topical given the COVID-19 situation and our evaluation, whilst not what we had originally planned, is highly relevant to meeting the needs of people with MS who are far more isolated than ever before. Pending our evaluation in this single-arm study, we propose a follow-on randomised feasibility trial (if major modifications are indicated during our evaluation) or a plan to progress to a full effectiveness evaluation with internal pilot to assess willingness to be randomised should only minor modifications be required.

## Data Availability

We aim to make our research data available wherever possible, subject to regulatory approvals, any terms and conditions placed upon us from external providers, patient confidentiality and all laws concerning the protection of personal information.

## Abbreviations

CTIMPs: Clinical Trials of Investigational Medicinal Products
COVID-19: Novel coronavirus (2019-nCoV)
DVD: Digital versatile disc
EDSS: Expanded Disability Status Scale
EQ-5D-5 L: 5-level (health-related quality of life outcome measure) by the EuroQol Group
GDPR: General Data Protection Regulation
HRA: Health Research Authority
LEAP-MS: Lifestyle, Exercise and Activity Package for people with Multiple Sclerosis
MFIS: Modified form of the Fatigue Impact Scale
MHRA: Medicines and Healthcare products Regulatory Agency
MS: Multiple sclerosis
MSIS-29: Multiple Sclerosis Impact Scale - 29
NHS: National Health Service
OxPAQ: Oxford Participation and Activities Questionnaire
PGIC: Patients’ Global Impression of Change
PwPMS: People with progressive MS
UW-SES: University of Washington 6-item short form self-efficacy scale
